# Risk attitude and risk perceptions of climate change among Indian cotton farmers

**DOI:** 10.1038/s41598-025-99125-2

**Published:** 2025-06-04

**Authors:** Reddyprasanna Diyyala, Qingxia Jenny Wang, Shahbaz Mushtaq, N. Venkatesa Palanichamy, D. Murugananthi, V. Geethalakshmi, M. Rajavel

**Affiliations:** 1 Centre for Applied Climate Sciences, University of Southern Queensland (UniSQ), Toowoomba, QLD Australia; 2 Agricultural and Rural Management, Tamil Nadu Agricultural University, Coimbatore, Tamil Nadu India; 3 School of Business, University of Southern Queensland (UniSQ), Springfield Education City, QLD Australia; 4 Agricultural College & Research Institute, Tamil Nadu Agricultural University, Coimbatore, Tamil Nadu India; 5 Directorate of Agribusiness Development, Tamil Nadu Agricultural University, Coimbatore, Tamil Nadu India; 6 Tamil Nadu Agricultural University, Coimbatore, Tamil Nadu India

**Keywords:** Risk perception, Risk attitude, Climate risk, Pests and diseases risk, Cotton farmers, Psychology and behaviour, Socioeconomic scenarios

## Abstract

Indian farmers have been facing significant production risks that have substantially decreased their potential yield. To reduce these risks, farmers employ various tactical strategies. The choice of strategy, however, depends mainly on their risk attitude and perceptions. This study examines the factors influencing farmers’ risk attitude and perceptions based on the survey of 350 cotton farmers from Virudhunagar district in Tamil Nadu, India. Risk attitude is evaluated using a Multiple Price List (MPL) experimental method, while the risk matrix is utilised to measure the risk perception of drought, uneven rainfall distribution, and pests and diseases. Logit model is applied to assess the variables associated with farmers’ risk attitude and perceptions. The findings reveal that 75% of the respondents exhibit risk-averse behaviour, while only 12% display a risk-seeking attitude. The majority of respondents identify drought and rainfall as major risks compared to pests and diseases. Logit model results show that gender, education, organisational membership, irrigation access, farming experience, access to credit, contact with extension personnel, and yield loss negatively influence farmers’ risk attitude. Similarly, organisational membership, a higher share of non-farm income, more farm size, irrigation availability, and credit accessibility reduce the farmers’ risk perceptions. These findings help policymakers understand how local farmers perceive farm risks such as drought, rainfall variations, and pests and diseases and consider these viewpoints when developing sustainable adaptation measures. This study highlights the significance of farmer group organisations, improved extension services, and credit access in shaping farmers’ risk attitude and perceptions, thereby enhancing farm productivity.

## Introduction

The decision-making domain in agricultural production systems presents a unique and complex environment, characterised by risks and uncertainties^1,2^. Farmers face a range of unpredictable factors, including weather patterns, pests, and diseases, which pose significant challenges and potentially impact their farm output. These risks may discourage farmers from engaging in high-return activities^3^. The economic impact of various risks on agricultural profitability depends partly on farmers’ willingness and ability to face risks^4^. Thus, the systematic management of risks associated with crop production is the fundamental aspect of ensuring the success of agricultural economies^5^.

To adapt to the risky environment, farmers make certain production decisions and employ various risk-mitigating strategies^6^. Many studies suggest that, in the face of risk and uncertainty, farmers’ risk attitude and perceptions are the major components in shaping their decision-making processes^3,7^ and choosing adaptation strategies at the farm level^1,8–10^. Risk perception is the subjective evaluation of the adverse impacts on human well-being or the environment resulting from a specific event or circumstance. Farmers’ risk attitude reflects their psychological feelings and interpretations of risk. Therefore, it is categorised as risk-averse, where individuals actively seek to minimise or avoid risk under all circumstances; risk-neutral, where individuals remain indifferent to both risk avoidance and risk-taking; and risk-loving, where individuals demonstrate a preference for engaging in risk^11,12^. Farmers’ risk-averse nature influences their adoption of modern technology^6,13^, which in turn significantly impacts the yield and farm income^1^. For instance, the risk-averse nature reduces the likelihood of adopting government-recommended improved varieties and preventive measures against pests and diseases^14^.

In addition, a study by Hasibuan, et al.^15^ found that farmers with a higher Risk Perception Index (RPI) for increasing temperature and precipitation were more likely to use chemical fertilizers and herbicides, while those with higher RPI for increasing rainy days tended to use more herbicides. By having a precise and timely perception of risk, farmers can evaluate the potential consequences and probability of risks and make informed decisions regarding crop management^16^ and coping mechanisms^10^. Therefore, it is crucial to understand farmers’ risk attitude and perceptions, and the factors influencing them. This enables accurate prediction of the decision-making process of households^7,17^.

This study focused on the risk attitude and perceptions of cotton farmers in the Virudhunagar district of Tamil Nadu, India. Over 60% of the global cotton supply is produced by smallholder cotton farmers^18^. In the case of developing countries, the major concern is the vulnerability of smallholder farmers to agricultural uncertainties and risks^1^ and their low capacity to absorb shocks^15^. It is a widespread practice to generalize the risk attitude of farmers worldwide, with smallholder farmers often being stereotyped as being excessively risk-averse. However, this assumption has been challenged with evidence from various studies that risk attitude differ across regions due to various cultural and institutional factors^1^. Despite facing similar risks in agriculture, farmers in the same region may have different perceptions and attitude towards it^19^.

Any policy aimed at enhancing agricultural production must prioritize the improvement of farmers’ risk management capabilities. Therefore, it is crucial for policymakers to understand how local farmers perceive a diverse range of risks associated with farming^1^. By considering these varying perspectives, policymakers can develop efficient and resilient strategic interventions that address the concerns of local farmers^3,19–21^. Thus, it is imperative to conduct in-depth research and analysis of factors that influence the risk behaviour of local communities towards crop production risks. Despite the importance of assessing farmers’ risk perceptions and attitude to better understand their risk management decisions, there have been no studies conducted in India to quantify these factors. Moreover, findings from studies beyond India may not reflect the behaviour of Indian cotton farmers. As a result, this research attempts to bridge this gap by gathering relevant empirical evidence on Indian cotton farmers to understand previous findings and draw reliable conclusions^1^.

Several subjective risk variables in explaining farmers’ risk perceptions provide evidence that farmers’ subjective risk perceptions deviate from the actual objective risk due to heterogeneity in their circumstances and personality traits^22^. Therefore, the main aim of this paper is to investigate how the risk attitude and risk perceptions of Indian cotton farmers are influenced by their personal and socioeconomic factors. Taking into consideration that risk attitude and risk perceptions are latent variables and cannot be observed directly, the extension services can utilise the knowledge of farmers’ personal and socioeconomic characteristics influencing risk attitude and risk perceptions to tailor risk management courses and workshops to specific farmers groups^23^.

This study provides a comprehensive understanding of how farmers perceive and manage risk. It also helps in accurately predicting the future choices and behaviour of Indian cotton farmers. Policymakers can use this information to develop practical policy tools and risk management strategies to assist farmers in dealing with potential losses from various sources of risks^1^. Furthermore, this study extends the scholarly understanding of cotton farmers’ risk perceptions and risk attitude. It sheds light on how these factors shape farmers’ experience of various risk impacts and decision-making processes^24^. By tailoring strategies to the unique needs of each community, we can promote long-term resilience in the face of uncertainty^19^.

The remaining part of the paper is structured as follows: Section 2 briefly describes the study area and sampling framework. Section 3 explains the empirical methodology and Section 4 presents the results and discussion. Finally, the paper ends with the conclusion, limitations, and scope for future research.

## Materials and methods

### Study area

This study was conducted in the Virudhunagar district of Tamil Nadu, India. The total geographical area of the district is 424,323 hectares, with a net cultivated area of 139,197 hectares. Of this, an area of 19,858 hectares is allocated to cotton cultivation. This region is located between 77° 28’ and 78° 50’ East longitude and 11° 00’ and 12° 00’ North latitude. The annual mean minimum and maximum temperatures in this district are 20.5 and 38.3 °C, respectively. This area faces a significant irrigation challenge due to its reputation for low rainfall and lack of a reliable river water source. Irrigation is predominantly sourced from rain-fed tanks. Only 57% of the area has access to guaranteed irrigation through wells, while the remaining areas rely entirely on rain-fed tanks to meet their irrigation needs.

The climate of this district is predominantly hot and arid for approximately nine months of the year, with the majority of rainfall occurring during the northeast monsoon. The cultivation of both food and non-food crops takes place in this district during two primary cropping seasons—*Kharif* and *Rabi*. The main food crops grown include paddy, maize, jowar, bajra, and various pulses, such as horse gram, black gram, and green gram. Additionally, cash crops like cotton and oilseeds such as groundnut are also cultivated, with cotton being the primary cash crop in this area (mostly during the *Rabi* season). The livestock sector in this region serves as a supplementary source of employment and sustainable income for small and marginal farmers^25^.

### Sampling framework

To achieve the objectives of the study, a multistage sampling technique combined with a simple random sampling technique was used to select the study area and the sampling farmers. The initial stage involved the selection of Virudhunagar district as the main study area from among 38 districts in Tamil Nadu. In the subsequent stage, Aruppukottai, Kariapatti, Rajapalayam, Sathur, and Virudhunagar blocks were selected out of 11 blocks in the selected district. In the next step, 5 villages were randomly selected from each block. Finally, households were randomly selected from each village using the below equation as suggested by Raza Ullah et al.^26^ and Akhtar, et al.^27^, resulting in a total sample size of 350 cotton households.

1 $$\:\text{n}=\:\frac{\text{N}}{{1+\text{N}\text{e}}^{2}}$$

where, n = Sample size in each village; N = Total number of farming households in a village; e = Precision which is set at 15% (0.15).

In Virudhunagar, cotton is primarily grown as winter rainfed and summer irrigated. However, the winter rainfed cotton is predominant, demonstrating the region’s resilience to climatic conditions. Sowing typically occurs between September and October, with a growth duration of 140 to 150 days. The survey was conducted between mid-September and mid-October 2022, and it involved interviewing 350 cotton farmers who were considered as household heads. These farmers had significant farming knowledge and were capable of making important financial decisions. Farmers were requested to provide data on their demographic and socioeconomic characteristics, as well as yield losses due to various risks encountered during October 2021 to March 2022, when cotton was cultivated. As drought, uneven rainfall distribution, and pests and diseases are the primary risks affecting farmers in this region, these parameters are included in the analysis. Yield losses related to these issues are assessed based on the farmers’ subjective self-reports of their losses.

## Empirical methodology

### Logit model

Logit model is used to analyse the factors influencing the risk attitude and risk perceptions of farmers. The dependent variables in this model are risk attitude and perception of two risk types i.e., climate risk (drought and uneven rainfall distribution) and biological risk (pests and diseases). The dependent variables are dichotomous, which takes values 0 or 1. Therefore, we used the binary logit model in this study. For risk attitude, a value of 1 is given to a risk-averse farmer and 0 otherwise^3,10,28^. Similarly, in the case of risk perception, a value of 1 is assigned to a farmer who has a high perception of risk and 0 otherwise^3,10,28^.

The logit model is specified as per Eq. (2):

2 $$\:{\text{y}}_{\text{i}}={\upalpha\:}\:+\:\sum\:_{\text{j}=1}^{\text{m}}{{\upbeta\:}}_{\text{j}}{\text{X}}_{\text{j}}+{{\upepsilon\:}}_{\text{i}},$$

where y_i_ is the independent variable and X_j_ is a vector of independent variables, which includes gender^28,29^, age^3,10,17,28,30^, education^10,28,30^, family size^3,10,17,28,30^, membership in the organization^31,32^, log of share of farm income^33^, log of share of non-farm income^3,10,28,30^, farm size^3,10,28^, source of irrigation, farming experience^3,10,28,30^, livestock^28^, access to credit^10,28^, exposure to mass media^3,34^, contact with extension personnel^3,30,34^, total yield loss, yield loss due to drought^33^, yield loss due to uneven distribution of rainfall and yield loss due to pests and diseases. $\:{{\upbeta\:}}_{\text{j}}$ is a set of estimated coefficients on the independent variables and $\:{{\upepsilon\:}}_{\text{i}}$ the error term. A detailed explanation of all variables used in the study is presented in Table 1.

**Table 1:** Description of variables used in the study.

Variables	Description
*Dependent variables*	
Risk attitude	1 if a farmer is risk-averse, 0 otherwise
Perception of drought	1 for high-risk perception and 0 for low-risk perception
Perception of uneven distribution of rainfall	1 for high-risk perception and 0 for low-risk perception
Perception of pests and diseases	1 for high- risk perception and 0 for low-risk perception
*Independent variables*	
Gender	1 for male and 0 for female
Age	Age of household head in years
Education	Level of education (1 = no schooling, 2 = primary school, 3 = middle school, 4 = secondary school, 5 = higher secondary, 6 = graduate, 7 = Postgraduate)
Family size	Number of members in a family
Membership in the organisation	1 if a farmer is a member of any organisation and 0 otherwise
Log of share of farm income	Logged value of % contribution of income from agriculture
Log of share of non-farm income	Logged value of % contribution of income from the sources other than agriculture
Farm size	Size of the farm in acres
Source of irrigation	1 for availability of irrigation source and 0 otherwise
Farming experience	Farming experience of household head in years
Livestock	1 for holding livestock and 0 otherwise
Access to credit	1 for access to credit and 0 otherwise
Exposure to mass media	Overall score of frequency of use of different media
Contact with extension personnel	Overall score of frequency of contacting each extension personnel
Total yield loss	Total yield loss occurred in the last year in %
Loss due to drought	Yield loss occurred due to drought in the last year in %
Loss due to uneven rainfall distribution	Yield loss occurred due to uneven rainfall distribution in the last year in %
Loss due to pests and diseases	Yield loss occurred due to pests & diseases in the last year in %

**Table 2:** The experimental design to determine the risk attitude.

Task	Option A	Option B	EV_a_	EV_b_	EV_a_ - EV_b_	CRRA interval
1	50	50(1/2);0(1/2)	50	25	25	-$\:\infty\:$, − 2.9
2	42	50(1/2);0(1/2)	42	25	17	− 2.9, − 1.1
3	36	50(1/2);0(1/2)	36	25	11	− 1.1, − 0.4
4	30	50(1/2);0(1/2)	30	25	5	− 0.4, 0
5	25	50(1/2);0(1/2)	25	25	0	0, 0.2
6	20	50(1/2);0(1/2)	20	25	− 5	0.2, 0.3
7	16	50(1/2);0(1/2)	16	25	− 9	0.3, 0.4
8	14	50(1/2);0(1/2)	14	25	− 11	0.4, 0.5
9	12	50(1/2);0(1/2)	12	25	− 13	0.5, $\:\infty\:$

*EV* Expected Value, *CRRA* Coefficient of Relative Risk Aversion.

### Determining the risk attitude of farmers

The risk attitude of farmers is measured using the Multiple Price List (MPL) experimental method based on the work of Holt and Laury^35^. It offers a series of lotteries with safe and risky options, which are presented in Table 2. Both options are characterised by two disbursement amounts. In option A, the probability of winning the amount remains the same, but the amount reduces systematically throughout the nine tasks. However, in option B, the amount and the probability of winning the amount remain the same. This experiment aims to identify the decision situation in which the farmers switch from a safe option to a risky option. Subjects are presented with nine decision scenarios and are required to point to the task at which they switched from option A to option B.

For each task, the value of the risk preference parameter is computed assuming that households have the constant relative risk aversion (CRRA) utility, as demonstrated in Eq. (3).

3 $$\:{\text{u}}_{\text{i}}=\frac{{\text{x}}^{1-\text{r}}}{1-\:\text{r}},$$

where x is the payoff in the option, and r is the coefficient of constant relative risk aversion (CRRA). A positive value of r signifies the risk-averse farmer, while zero and negative r value indicates a risk-neutral and risk-seeking farmer, respectively. For simplification, we used 0 for a risk-taking or risk-neutral farmer and 1 for a risk-averse farmer, in the logit analysis.

### Measuring the risk perception of farmers

**Fig. 1:**
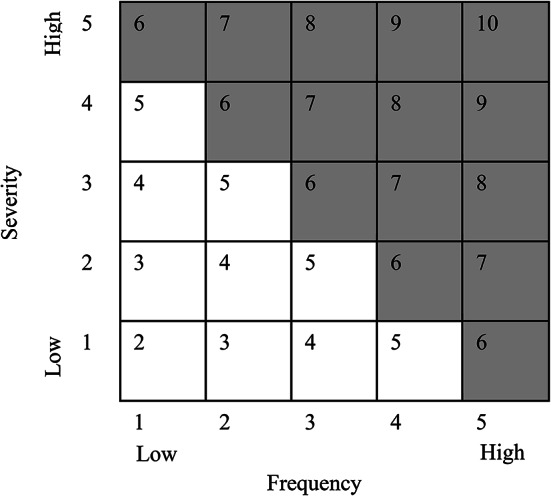
The risk matrix.

Risk perceptions of cotton farmers are analysed using a risk matrix, which is shown in Fig. 1. Farmers are asked to score the severity and frequency of various risks, such as drought, uneven distribution of rainfall, and pests and diseases, which they encountered in the past decade. A Likert scale is employed to determine the impact of these risks on production, with a range of 1 to 5 representing two to ten years for the frequency of risk events [(0–2)1, (3–4)2, (5–6)3, (7–8)4 and (9–10)5]. For severity, 1 denotes low severity and 5 indicates high severity. The score of frequency and severity of each risk are combined on the risk matrix, following Ogurtsov, et al.^36^ and Iqbal, et al.^10^. If the total score of a specific risk falls between 2 and 5, it is considered to be low, and if the score ranges from 6 to 10, it is categorised as high. While performing the logit analysis, a value of 1 is assigned to a farmer who has a high perception of risk and 0 otherwise.

**Fig. 2:**
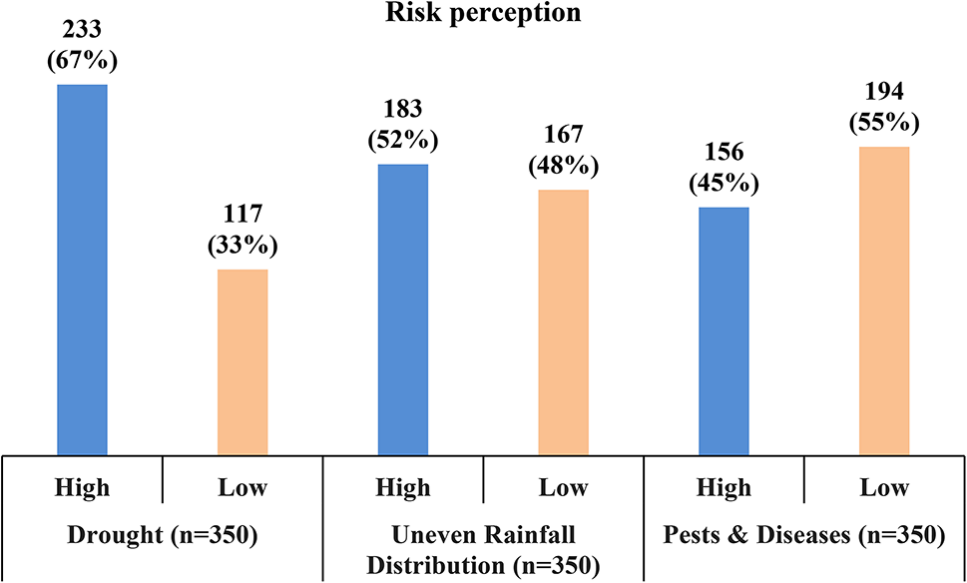
Classification of risk perception of farmers.

## Results and discussion

### Socioeconomic characteristics of farmers

The data presented in Table 3 provides descriptive statistics on the socioeconomic backgrounds of the farmers who were surveyed. It is observed that the majority of the participants are males, accounting for 84% of the sample, with an average age of 49 years. The level of education among the respondents is found to be equivalent to middle school which ranges from 6th to 8th grade. However, the highest level of education found among the surveyed farmers is post-graduation. In addition, over 41% of the individuals having membership in the organization. This limited participation suggests that access to institutional support and risk management resources is not widespread. Organisational members may develop more informed risk perceptions and structured risk management approaches, while non-members, lacking these resources, may exhibit greater risk aversion.

The households are found to have a family with an average of four members. The farmers earn an average annual income of approximately Rs.337,000 from their farming activities and an additional Rs. 136,462 from non-farm sources. The diversification of income is often regarded as a strategy for managing risk. This approach indicates that farmers who earn a significant portion of their income from non-agricultural activities may perceive agricultural risks as being greater. Consequently, they tend to explore supplementary income opportunities as a means of mitigating these risks. Furthermore, the results show that the maximum income generated from farming activities is Rs.200,00, while the maximum income earned from non-farm activities is Rs.720,000. The farms varied in size from 1 to 30 acres, with the mean farm size of the households being around 5.7 acres. Only 38% of the sample has access to irrigation. The average years of farming experience is approximately 26 years, ranging from a minimum of 1 year to a maximum of 51 years. Nearly half of the sampled farmers (47%) are engaged in livestock farming, which serves as a supplementary source of income and may influence their risk attitudes. Furthermore, 60% of the participants indicated that they have access to credit, which could shape their perception of financial risk and their approach to risk management strategies.

### Risk attitude and risk perceptions of farmers

In the agriculture sector, comprehending the farmers’ risk preferences is paramount to gaining valuable insights into their decision-making processes regarding risk management strategies and behaviour under risk. There are different types of risk attitude among farmers that significantly affect how they manage it. The risk attitude of farmers is categorized based on the r-value calculated using Holt and Laury^35^ MPL (Multiple Price Lists) experimental methods. A positive r value indicates a risk-averse farmer, while zero and a negative r value indicate a risk-neutral and risk-seeking farmer, respectively. The results are presented in Table 4, showing that a vast majority of the respondents (75%) are risk-averse, with a significant proportion expressing a very high level of risk aversion (36%). Only a small percentage of farmers (12%) are risk seekers, with a slight to moderate inclination to take risks.

To implement a practical and targeted risk management system, it is crucial to have a thorough comprehension of the potential risk factors. The subjective risk perceptions of respondents are classified as high and low using the risk matrix depicted in Fig. 1. Farmers’ risk perception of drought, uneven distribution of rainfall, and pests & diseases are presented in Fig. 2. The findings reveal that approximately two-thirds of the respondents (67%) perceive drought as a high-risk factor, which indicates that they are cognizant of the impact of water scarcity on their crop yield. Moreover, over half of the respondents (52%) have a high-risk perception of the uneven distribution of rainfall, which suggests their understanding of the effect of climate change on weather patterns. However, in the case of pests and diseases risk, only 45% of farmers show a high-risk perception. Overall, these findings demonstrate that farmers have increased risk perception towards climate change compared to pests & disease outbreaks, which could be an opportunity to implement proactive approaches to deal with climate risks.

**Table 3:** Descriptive statistics of farmers’ socioeconomic characteristics.

Variables	Mean	SD	Minimum	Maximum
Gender	0.84	0.37	0	1
Age	49.08	8.88	25	68
Education	2.88	1.42	1	7
Family size	4.19	1.59	1	13
Membership in organisation	0.41	0.49	0	1
Farm income (in Rs.)	337,811	196,844	50,000	200,000
Non-farm income (in Rs.)	136,462	141,947	1000	720,000
Farm size	5.71	3.78	1	30
Source of irrigation	0.38	0.49	0	1
Farming experience	25.51	11.57	1	51
Livestock	0.47	0.50	0	1
Access to credit	0.60	0.49	0	1

**Table 4:** Classification of farmers based on their risk attitude.

Category	Particulars	Frequency (*n* = 350)	%
Risk-seeker	Very high risk-seeker	0	0.00
	High risk-seeker	0	0.00
	Moderate risk-seeker	9	2.57
	Slight risk-seeker	33	9.43
Risk-neutral	Risk-neutral	46	13.14
Risk-averse	Slightly risk-averse	27	7.71
	Moderately risk-averse	59	16.86
	Highly risk-averse	51	14.57
	Very highly risk-averse	125	35.71
Total		350	100

**Table 5:** Logit estimates of the determinants of risk attitude.

Variables	Risk attitude	
	Coefficients	Marginal effects
Gender	− 1.268^**^ (0.594)	− 0.142^**^ (0.065)
Age	0.032 (0.027)	0.004 (0.003)
Education	− 0.545^***^ (0.133)	− 0.061^***^ (0.014)
Family size	0.050 (0.107)	0.006 (0.012)
Membership in the organisation	− 1.082^***^ (0.353)	− 0.121^***^ (0.038)
Share of farm income	0.437 (0.753)	0.059 (0.084)
Share of non-farm income	− 0.296 (0.320)	− 0.033 (0.036)
Farm size	0.019 (0.052)	0.002 (0.006)
Source of irrigation	− 0.647^**^ (0.330)	− 0.073^**^ (0.037)
Farming experience	0.047^**^ (0.021)	0.005^**^ (0.002)
Livestock	0.218 (0.335)	0.024 (0.037)
Access to credit	− 1.077^***^ (0.373)	− 0.121^***^ (0.042)
Exposure to mass media	0.028 (0.097)	0.003 (0.011)
Contact with extension personnel	− 0.143^*^ (0.073)	− 0.016^*^ (0.009)
Total yield loss	0.040^***^ (0.013)	0.004^***^ (0.001)
Log likelihood	− 125.11	
LR Chi^2^ (15)	144.51	
Prob > Chi^2^	0.000	
Pseudo R^2^	0.37	
Number of observations	350	

***, * and * indicates significance level of 1%, 5% and 10% respectively. Numbers in parentheses are standard errors.

**Table 6:** Logit estimates of the determinants of risk perception.

Variables	Risk perception					
	Drought		Uneven rainfall distribution		Pests and diseases	
	Coefficients	Marginal effects	Coefficients	Marginal effects	Coefficients	Marginal effects
Gender	− 0.539 (0.454)	− 0.053 (0.044)	0.282 (0.373)	0.045 (0.059)	− 0.437 (0.366)	− 0.069 (0.057)
Age	0.217^***^ (0.033)	0.021^***^ (0.002)	0.091^***^ (0.024)	0.014^***^ (0.004)	− 0.111^***^ (0.025)	− 0.018^***^ (0.004)
Education	− 0.020 (0.134)	− 0.002 (0.013)	0.189^*^ (0.107)	0.030^*^ (0.017)	0.667^***^ (0.116)	0.105^***^ (0.015)
Family size	− 0.193 (0.130)	− 0.019 (0.013)	0.052 (0.091)	0.008 (0.014)	0.075 (0.090)	0.012 (0.014)
Membership in the organisation	− 0.695^**^ (0.358)	− 0.069^**^ (0.035)	− 2.218^***^ (0.314)	− 0.351^***^ (0.035)	0.244 (0.289)	0.038 (0.046)
Share of farm income	− 1.029 (0.770)	− 0.102 (0.075)	− 0.988 (0.643)	− 0.156 (0.101)	0.717 (0.634)	0.113 (0.100)
Share of non-farm income	0.555^**^ (0.264)	0.055^**^ (0.025)	− 0.610^**^ (0.251)	− 0.097^**^ (0.039)	0.139 (0.219)	0.022 (0.034)
Farm size	− 0.154^***^ (0.058)	− 0.015^***^ (0.005)	− 0.270^***^ (0.061)	− 0.043^***^ 0.009	0.177^***^ (0.055)	0.028^***^ (0.008)
Source of irrigation	− 2.316^***^ (0.418)	− 0.228^***^ (0.033)	0.198 (0.292)	0.031 (0.046)	0.278 (0.291)	0.044 (0.046)
Farming experience	0.055^**^ (0.023)	0.005^**^ (0.002)	− 0.014 (0.017)	− 0.002 (0.003)	0.015 (0.018)	0.002 (0.003)
Livestock	− 0.130 (0.347)	− 0.013 (0.034)	0.027 (0.276)	0.004 (0.044)	− 0.308 (0.281)	− 0.049 (0.044)
Access to credit	− 0.445 (0.371)	− 0.044 (0.036)	− 0.959^***^ (0.301)	− 0.152^***^ (0.045)	− 0.255 (0.283)	− 0.040 (0.045)
Exposure to mass media	− 0.054 (0.106)	− 0.005 (0.010)	0.040 (0.085)	0.006 (0.013)	− 0.089 (0.085)	− 0.014 (0.013)
Contact with extension personnel	0.078 (0.071)	0.008 (0.007)	0.106 (0.067)	0.017 (0.011)	0.294^***^ (0.106)	0.046^***^ (0.016)
Loss due to drought	0.025^*^ (0.014)	0.002^**^ (0.001)				
Loss due to uneven rainfall distribution			0.007 (0.011)	0.001 (0.002)		
Loss due to pests and diseases					0.042^*^ (0.014)	0.007^*^ (0.002)
Log likelihood	− 109.57		− 168.93		− 167.77	
LR Chi^2^ (15)	226.88		146.60		145.53	
Prob > Chi^2^	0.00		0.00		0.00	
Pseudo R^2^	0.51		0.30		0.30	

***, * and * indicates significance level of 1%, 5% and 10% respectively. Numbers in parentheses are standard errors.

### Factors influencing risk attitude and risk perceptions of farmers

The findings of the logit analysis are displayed in Tables 5 and 6. The pseudo-R^2^ served as a measure of the logit model fit to data. When the pseudo-R^2^ value is 1, it signifies a good model fit, while 0 indicates no relationship. A pseudo-R^2^ greater than 0.2 is indicative of a relatively good fit^37^. All the pseudo-R^2^ values in these models are greater than 0.2, indicating a good model fit. When using logistic regression to fit models, it is also crucial to consider the possibility of multicollinearity among independent variables. Two important indicators for detecting multicollinearity are Tolerance (TOL) and the variance inflation factor (VIF). If the TOL value is less than 0.2 or the VIF value is greater than 10, it may indicate serious multicollinearity among independent variables. However, in all the models studied, the TOL value is greater than 0.2 and the VIF value is less than 10, which suggests that multicollinearity is not a significant concern in these models.

#### Determinants of risk attitude

Results presented in Table 5 reveal that variables such as gender, education, membership in the organisation, source of irrigation, access to credit and contact with extension personnel are negatively and significantly correlated with the risk attitude of farmers. However, farming experience and yield loss are positively and significantly correlated with farmers’ risk attitude.

The influence of gender on farming decisions is a significant factor that reflects the division of labour prevalent in farming systems^38^. It is clear from Table 5 that gender has a significant negative effect on the probability of being risk-averse. Specifically, being male increased the probability of being willing to take risks by 14.2%. This finding is understandable because men tend to be more socially interactive and better at managing risk. Similar results were found in previous studies^28,39–42^. In contrast, Addey^43^ concluded that males in Ghana are burdened with many responsibilities, reducing their probability of being risk-seeking but increasing their risk aversion to being risk-neutral.

Results show that farmers who possess higher education levels tend to have a greater propensity for risk-seeking^30^. This could be because education expands the farmers’ ability to acquire and utilize knowledge related to adaptive strategies against various sources of risks and protects their earnings from various hazards^3,30,44^ while offering alternative job opportunities for them^38^. It is found that farmers with higher levels of education are 6.1% more likely to take risks. This finding aligns with previous studies conducted by Velandia, et al.^45^, Bocquého, et al.^46^, Zeweld, et al.^38^, and Farhan, et al.^30^, but contradicts Roe^42^, Ahmad, et al.^28^ and Ahmad, et al.^29^ who concluded that farmers with more formal education are less risk tolerant than with less than a high school degree.

It is worth noting that there appears to be a notable inverse relationship between farmers’ membership in an organization and their attitude towards risk. In particular, farmers who are members of an organization are more likely to be comfortable with risk, with a 12.1% increase in the likelihood of being willing to take risks. This outcome can be attributed to the economic benefits of social participation, including enhanced cooperation and improved access to technical knowledge and information^31^.

The results also show that the availability of a reliable source of irrigation can significantly increase a farmer’s willingness to take risks by 7.2%. This is because irrigation provides farmers with the opportunity to diversify their crops and expand their agricultural enterprises^6^. By diversifying their crops, farmers can mitigate the potential losses of one enterprise with the profits of another. It not only increases their chances of success but also allows them to make more informed decisions and take calculated risks.

The positive and significant coefficient of farming experience indicates that farmers with more experience in farming tend to take less risk compared to the less experienced farmers. In other words, the probability of farm households’ willingness to take risks decreases by 0.5% with a unit increase in farming experience. This finding is likely due to the fact that experienced farmers possess a superior understanding of various production risks and potential losses associated with risk-taking. Furthermore, their extensive knowledge enables them to exercise greater caution and avoid unnecessary risks^30^. This finding is similar to Ahmad, et al.^29^ and Farhan, et al.^30^, but different from Ahmad, et al.^28^ and Islam, et al.^34^.

Access to credit exhibits a notable inverse association with risk aversion, which is similar to the findings of Islam, et al.^34^ and Dadzie and Acquah^47^, but contradicts Ahmad, et al.^29^. Results indicate that farmers with access to credit are 12.1% more likely to take risks. It is due to the fact that access to credit allows farmers to manage their farm risk as credit can be utilized as an ex-post risk management tactic, minimising their concern about potential sources of risk^3^. Additionally, extending financial support to farmers can enhance their productivity by improving their access to technological learning and production inputs^47^.

It is also observed that farmers who maintain consistent contact with extension personnel exhibit a greater propensity for risk-taking behavior. This finding is supported by Akhtar, et al.^7^ and Fahad, et al.^48^. Results show that farmers who have more access to extension officers are 1.6% more willing to take risk. The reason for this may be that access to extension officers enables farmers to adopt proper risk management tools and manage various farm risks, which ultimately leads to improved farm productivity^7,48^.

Furthermore, research indicates that when farmers face yield loss, they tend to become more risk-averse in their subsequent decision-making. Specifically, the findings indicate that farmers who have experienced yield loss in the previous year are 0.4% more likely to be risk-averse. The experience of crop yield reduction due to various risks can significantly impact the income and livelihood of farmers. As a result, they may become more cautious about taking risks in order to maintain a steady income, which makes them more risk-averse.

#### Determinants of risk perception

Table 6 presents the factors that influence the risk perceptions of cotton farmers related to drought, uneven rainfall distribution, and pests and diseases. Previous studies have reported inconclusive results concerning the impact of households’ personal and socioeconomic characteristics on the risk perceptions of farmers. This study has also revealed mixed results concerning the impact of household socioeconomic characteristics on the perception of various risks among cotton farmers. In the case of drought risk perception, the variables such as age, share of off-farm income, and yield loss due to drought show a significant positive correlation, while membership in the organisation, farm size, and access to irrigation are negatively associated. Similarly, risk perception of rainfall is found to be significantly and positively related to age and education and negatively correlated with membership in the organisation, share of non-farm income, farm size, and access to credit. For pests & diseases risk, only age exhibited a negative correlation with risk perception, whereas education, farm size, contact with extension personnel, and yield loss due to pests & diseases displayed a positive association.

Among the various independent variables influencing the risk perception, age has a mixed influence on farmers’ perception of various. Results show that as the farmers’ age increases, the perception of drought and rainfall risk is high, which is consistent with Farhan et al.^30^. This could be because older farmers mostly relied upon indigenous technical knowledge against climate adaptation, while younger farmers supported them through digital weather information services. However, in the case of pests and diseases, results indicate that young farmers have a high-risk perception, which aligns with research conducted by Akhtar, et al.^7^.

Findings show that education plays a crucial role in developing appropriate perception of rainfall risk^29,49–51^ and pests and diseases risk^29,51^. Specifically, as the level of education increases, farmers are 3.0% more likely to view rainfall as a major risk and 10.5% more likely to consider pests and diseases as a high-risk event. Educated farmers may have the ability to access global, regional, and country-level information about the impacts of climate change^49^ and the occurrence and severity of insects/pests, which raises their perceptual level^30^. Therefore, more educated farmers may have a high-risk perception compared to their counterparts. However, some studies argued that educated farmers are likely to possess the necessary knowledge and skills to manage various risks efficiently, which lowers risk perception, and thus has a negative effect on risk perception of rainfall^10^ and pests and diseases^10,52^.

Membership in the organisation has a significant negative association with the perception of drought and rainfall risk. Findings show that farmers who were members of the organization are 6.9% less likely to perceive drought risk and 3.5% less likely to perceive rainfall as a high risk. These results further strengthen the argument that cooperative membership is an optimal means for promoting both the economic and social development of the farmer^50^. Thus, organisational membership can enable farmers to better prepare for potential challenges and risks by enhancing their awareness and knowledge levels for better climate adaptation and mitigation strategies^32^.

Generally, non-farm income is more stable than income from farming, and it helps to reduce the overall instability of the household’s income during times of uncertainty^53^. Similarly, the results show that the perception of rainfall risk tends to be lower when a household has a higher contribution of off-farm income. However, in the case of drought, a higher share of non-farm income is associated with an enhanced perception of drought risk, which is similar to Kaczała^33^, but contradicts Ahmad, et al.^29^. It may be plausible because non-farm income sources may not always be adequate in ensuring long-term sustainability and may instead engender a sense of high drought risk.

The size of a farm has a notable impact on how farmers perceive the risk. Farm size is negatively and significantly related to drought and rainfall risk perception. Farmers with large farm holdings determined drought and rainfall to be less risky. In fact, households with more farm size are 1.5% less likely to perceive drought risk and have a 4.3% lower probability of perceiving rainfall risk. This could be because farmers with large landholdings have greater wealth, more stability in income flow, and a larger asset base^16^ which makes them capable of diversifying farm activities and reducing stress about the consequences of any risk^54^. In addition, large farmers may have more resources to invest in technology or infrastructure and access to various risk management tools, such as crop insurance, that can help mitigate the impact of climate^48^.

However, in the case of pests and diseases, farm size shows a positive relation with risk perception, which is inconsistent with Ahmad, et al.^28^ and Ahmad, et al.^29^. The results indicate that the likelihood of perceiving risks associated with pests and diseases increases by 2.8% with an increase in farm size. It may be possible because farmers may opt for crop diversification to mitigate the risks, particularly those who engage in intercropping to increase on-farm activities. Such farmers perceive risks more frequently because they choose crops with varying maturation periods which may attract birds, wild animals, pests, and diseases as hosts over a longer period, resulting in a high risk of disease^54^.

Irrigation source availability is associated with a lower perception of drought risk among farmers. Findings revealed that the probability of farmers’ perception of drought risk reduces by 22.8% with the availability of irrigation sources. This could be possible because farmers with access to irrigation sources can ensure a consistent water supply to cultivate crops throughout the year, even during periods of deficit rainfall. It helps to avoid crop losses and improve the volume of production, ultimately safeguarding the livelihood of farmers. This result is similar to Ndamani and Watanabe^49^, whose study concluded that resource-poor farmers are more likely to observe and feel the impacts of extreme climate change events since rainfed agriculture is their main source of livelihood.

In addition, the study reveals that farming experience influences drought risk perception. Findings indicate that farmers with more farming experience are 0.5% more likely to perceive drought risk. According to Duinen, et al.^22^ and Kaczała^33^, farmers’ risk perception of drought is a reliable indicator of their exposure and sensitivity to drought risk. Therefore, experienced farmers who have been frequently exposed to and affected by droughts know the consequences in terms of yield loss, which may lead to perceiving drought as a potential threat.

Credit availability has a negative and statistically significant association with the perception of rainfall risk, which is in line with Ahmad, et al.^28^. This means that households with access to credit tend to perceive low rainfall risk. Farmers who have access to credit are 15.2% less likely to perceive rainfall as a high-risk event. By providing farmers with access to credit sources, they become better equipped to manage risk in the event of a natural disaster. The borrowed funds can be invested in diversifying crops and income, which enables them to maintain a steady income and perceive low risk^16^.

It is surprising to note that contact with extension personnel increases the pests and disease risk perception among farmers. The results show that farmers with access to contact extension personnel have a 4.6% higher probability of considering pests & diseases as a potential risk source. This finding is consistent with the research by Farhan, et al.^30^ which demonstrates that although access to extension services helps farmers to understand different types of crop pests and diseases, it is possible that the limited efficacy in the control of pests and diseases, despite increased extension contacts, leads to a high level of risk perception.

Furthermore, the results show that farmers who have suffered from crop losses due to drought and pests and diseases infestation in the previous year, tend to exhibit a heightened sense of drought and pests and diseases risk perception. This is attributed to the uncontrollable effects of drought and pests and diseases, which leads to a significant yield loss. As per the findings of Kaczała^33^, the magnitude of risk perception associated with severe loss due to infrequent events is more pronounced than that associated with minor losses resulting from frequent events. Therefore, it can be concluded that farmers in this study may have encountered substantial crop losses in the previous year due to drought and pests and diseases, identified these factors as critical threats to farming activities.

## Conclusion and recommendations

Farmers’ decisions are significantly influenced by their attitude towards risk and their perception of uncertainty. It is, therefore, essential to comprehend the risk behaviour of decision-makers to ensure that farm decisions are productive and to assess the impact of policies accurately. An improved understanding of how farmers’ risk behaviour changes with socioeconomic characteristics and how these variations affect their decision-making, could help in comprehending demographic disparities. Additionally, this will enable a more informed decision-making approach and help to mitigate potential risks.

This study findings indicate that most of the farmers tend to be risk-averse while only a small proportion are risk-seeking. Many farmers are more concerned about the risks associated with drought and uneven rainfall distribution when compared to the risks posed by pests and diseases. Results from the logit analysis show that farmers who are males, have higher levels of education, membership in the organization, access to credit, access to irrigation, and contact with extension workers are more willing to take the risk. It is also found that farmers who have membership in the organisation, more farm size, and access to irrigation view the drought as minimal risk. Similarly, the risk perception of rainfall tends to reduce with having membership in the organisation, a larger share of non-farm income, more farm size, and access to credit.

These findings provide useful insights into farmers’ behavioural tendencies in response to their socioeconomic factors and allow policymakers to accurately anticipate the impact of policies on the farming community. This study emphasizes the importance of social factors such as education and organisational membership in influencing economic decision-making. In addition, the potential of developing farmer group organisations/farmer cooperatives to enhance formal extension services is highlighted, with guidance from the government or related industries. Formal organisations enable rural people to establish strong links with local community groups and institutions and encourage joint action and decisions to reduce the risk. Moreover, proper information access through enhanced extension services can transform the farmers’ risk attitude and enable them to choose appropriate risk management tools to improve farm productivity. Furthermore, providing access to microfinance institutions can allow farmers to better manage risk and encourage them to invest in new technologies and equipment, which in turn enhance crop production efficiency.

However, this study is limited in scope due to the small sample size and focus on only one district. To better understand the challenges and opportunities facing the cotton industry, future studies can be expanded to incorporate a larger sample size with the coverage of other cotton-growing areas. In addition, the study did not examine how closely farmers’ personal risk perceptions align with the actual estimates of risk in terms of the expected damages and probabilities. Therefore, more research is needed to determine the extent to which farmers’ risk perceptions accurately reflect the actual estimates. In this study, Holt and Laury (2002) experimental method is implemented using small stakes to facilitate farmers’ understanding of the design. However, to enhance the accuracy of decision-making, the use of higher payoffs is recommended. It is also recommended that future research incorporate factors such as traditional knowledge, social norms, market dynamics, and cultural influences. Understanding these elements is essential, as they may significantly shape farmers’ perceptions and behaviors. Furthermore, the study also proposes the analysis of the impact of risk attitude and risk perceptions on the adoption of various risk management strategies among cotton farmers. This information can enable stakeholders to encourage farmers to adopt robust risk management measures.

## Data Availability

The datasets used and/or analyzed during the current study are available from the corresponding author upon reasonable request.
